# Megestrol Acetate-Induced Symptomatic Hypogonadism in a Male Patient

**DOI:** 10.1155/2018/7048610

**Published:** 2018-07-18

**Authors:** Lubna Bashir Munshi, Yumiko Tsushima, Kwan Cheng, Maria Brito

**Affiliations:** ^1^Division of Endocrinology, Diabetes, and Bone Disease, Icahn School of Medicine at Mount Sinai, Mount Sinai Beth Israel, New York, NY, USA; ^2^Department of Internal Medicine, Mount Sinai Beth Israel, New York, NY, USA

## Abstract

The hypothalamic-pituitary-adrenal (HPA) axis and the hypothalamic-pituitary-gonadal (HPG) axis are very sensitive and can be affected by external factors like stress, starvation, and medication. Medication-induced suppression of these axes can cause adrenal insufficiency (AI) and hypogonadism. Exogenous glucocorticoid use is the most common cause of iatrogenic AI. Our aim is to bring attention to another broadly prescribed medication, megestrol acetate (MA), as the cause of suppression of both these axes. We report a case of symptomatic hypogonadism and asymptomatic AI in a male patient secondary to MA. The patient presented with decrease in testicular size and erectile dysfunction. His total testosterone and morning cortisol levels were low, but FH, LH, and TSH were normal. His pituitary MRI was unremarkable. Upon discontinuation of MA, the patient's testosterone and cortisol levels normalized and his symptoms resolved. Hypogonadism and AI are known adverse effects of MA, but symptomatic hypogonadism as the primary manifestation has only been reported once in previous literature. Prolonged hypogonadism can lead to sarcopenia, depression, and osteoporosis, while asymptomatic AI carries the risk of becoming overt AI. Thus, heightened awareness of the impact of MA on both these axes is necessary.

## 1. Introduction

MA is a synthetic progestin that has been in use since the 1970s to treat anorexia and cachexia mainly associated with cancer and HIV/AIDS. While its effectiveness in weight gain has been demonstrated in a previous review [1], several studies have reported adverse effects of MA such as hyperglycemia, diabetes, Cushing's syndrome [2], adrenal insufficiency (AI), and hypogonadism [3, 4]. The potentially fatal outcome of AI and severe long-term consequences of untreated hypogonadism are concerning and warrant consideration. We describe a case of symptomatic hypogonadism with asymptomatic AI in an HIV-infected man receiving MA.

## 2. Case

A 48-year-old male with past medical history of hyperlipidemia, HIV, and latent secondary syphilis presented for evaluation of loss of libido and erectile dysfunction for 2 months' duration. He had no other complaints. On examination, the patient was hemodynamically stable and did not show any signs of adrenal insufficiency. The only remarkable physical finding was a decrease in bilateral testicular size. On lab work, total testosterone level was 21.47 ng/dL (N: 300-1080 ng/dL) and morning cortisol was <1.0 *μ*g/dl. (N: 6.7-22.0 *μ*g/dL). Luteinizing hormone (LH), follicular stimulating hormone (FSH), and thyroid-stimulating hormone (TSH) as well as serum electrolytes were within normal limits. Upon reviewing his medication list, we found that the patient was taking MA (Megace) 800 mg daily as an appetite stimulant.

About 1 month prior to starting this medication, his total testosterone was normal at 548 ng/dl (N: 262-1593 ng/dl) along with his FSH, LH, prolactin, prostate specific antigen, and sex hormone binding globulin. Brain MRI showed only a partial empty sella and no other abnormalities. After excluding all other potential causes, MA was deemed to be responsible for his secondary hypogonadism. The patient was advised to taper down his MA slowly over a period of 6 weeks. Upon tapering down MA, the patient immediately showed improvement of his symptoms. His repeat lab work 4 weeks after discontinuation of MA revealed total testosterone, 798 ng/dl (N: 300-1080 ng/dl), and random cortisol, 6.0 *μ*g/d. (N: 2.0-14.0 *μ*g/dL). His libido returned, testicular size showed improvement, and he started to experience normal erections. He was started on an alternative appetite stimulant and is currently doing well.

## 3. Discussion

MA is an appetite stimulant used in patients with anorexia and cachexia mainly secondary to cancer and HIV/AIDS. Its glucocorticoid-like properties and potential to cause AI have been well described, but its potential to cause hypogonadism is not widely recognized. MA is known to have glucocorticoid-like properties with 46% affinity for glucocorticoid receptors compared to cortisol's 25% [5]. Due to this high affinity, MA has been associated with symptoms such as hyperglycemia, diabetes, and Cushing's syndrome [2]. Thromboembolism, alopecia, and impotence have also been reported [3]. AI is another adverse effect of MA which has been discussed in the literature. Our review found several reports of MA-induced symptomatic AI as the presenting feature of MA toxicity [3, 4, 6, 7]. Interestingly, AI does not necessarily occur during tapering or after discontinuation of MA but is seen in patients who are actively taking full doses. MA appears to have conflicting properties of both glucocorticoid activity and suppression, but the pathophysiology of MA-induced AI is unclear. Mann et al. hypothesized that this finding may be due to patients' incompliance with the medication, a state of stress overworking the already suppressed HPA axis, dual agonist and antagonist properties of MA (weak agonist to glucocorticoid receptors and antagonist to more potent endogenous glucocorticoids), or a stronger ability of MA to suppress the HPA axis than exerting peripheral glucocorticoid activity [2].

In our case, HPA was likely suppressed as evidenced by the undetectably low morning cortisol, but the patient did not have any physical signs or symptoms of AI. In a study by Leinung* et al.*, all of the 3 participants had confirmed HPA axis suppression without overt symptoms of AI [6]. This shows that biochemical evidence of HPA axis suppression does not necessarily cause symptoms. A trigger, such as an infection, may be needed to cause a state of acute stress in order to produce overt AI. Furthermore, all participants of Leinung et al.'s study had worsened glucose tolerance while receiving MA [6]. The coexistence of glucose intolerance and HPA axis suppression reinforces the finding that MA has both peripheral glucocorticoid activity and HPA axis suppression properties. MA toxicity causes both AI and hypercortisolism to some extent at baseline but which one of them is manifested and appears more predominantly depends on individual patient factors. Patient factors may be preexisting glucose intolerance or drugs that may interact with MA or the HPA axis. There may even be genetic differences that have yet to be discovered, which predispose an individual to have stronger or weaker glucocorticoid activity with MA use. Exogenous factors can be any insult that has the potential to trigger a stress response in a patient such as major surgeries, burns, and severe infections. Given the inherent glucocorticoid activity and HPA axis suppression of MA, precautions must be taken in order to avoid overt AI. MA should never be abruptly discontinued but rather tapered and stress dose steroids must be considered in situations where profound stress is anticipated or has occurred.

Hypogonadism is also a known adverse effect of MA [3, 4]. However, there are fewer reports of symptomatic hypogonadism compared to AI possibly because hypogonadal symptoms can be masked by AI symptoms. Although hypogonadism may not present as acutely as AI or cause death, it is a condition that must be monitored as there are long-term consequences. Our literature search only revealed one case report of symptomatic hypogonadism as the primary manifestation of MA toxicity [8].

Hypogonadism is relatively common in HIV-infected men with prevalence of 13%-40% in those treated with HAART. The pathophysiology is still unclear but it is likely due to multiple factors including infection of the pituitary, effects of antiretroviral drugs, effects of opiates, and poor health status. Clinical manifestations of androgen deficiency are hot flashes, reduction in testicular volume, decreased erections, body hair loss, and nonspecific symptoms such as depression, fatigue, osteoporosis, increase in visceral fat, and reduced lean muscle mass [9]. Hot flashes and reductions in erections are easy to detect but osteoporosis, depression, and loss of muscle mass are insidious conditions that can have detrimental effects on functional status and quality of life in the long term. Therefore, it is crucial to address hypogonadism early and be aware of high-risk populations. There have been several hypotheses regarding the pathophysiology behind MA-induced hypogonadism. Bodenner et al. reported a 50% decrease in LH levels in their study after administration of MA [10]. Hyperprolactinemia, which is also associated with hypogonadism, was noted in 19 of 21 subjects with a 150% rise in prolactin levels in the same study. Others have documented that MA decreases the number of androgen receptors (Geller et al.) [11]. However, these hypotheses are still under investigation and the exact mechanism of MA-induced hypogonadism is unknown.

MA's effectiveness as an appetite stimulant was demonstrated in the 2017 Cochrane review [1], although its mechanism is still unknown. Unfortunately, weight gain due to MA has been reported to be mostly fat mass and not muscle mass. Despite significant weight gain with MA use, there was loss of skeletal muscle mass and an increase in fat mass in a study done by Lambert et al. [12]. This leads to concerns about the clinical utility of MA-induced weight gain, since loss of muscle mass is not beneficial for improving functional status. Given that HIV-infected men have higher prevalence of hypogonadism, they are already at increased risk of developing sarcopenia. Therefore, MA use in this population requires careful consideration of the individual's risks before initiation as well as close monitoring of those receiving it.

According to the Endocrine Society guidelines, the following conditions warrant testing for low testosterone levels in men: sellar mass, treatment with medications affecting testosterone metabolism such as steroids and opioids, HIV-associated weight loss, end-stage renal disease, maintenance dialysis, moderate-to-severe chronic obstructive lung disease, infertility, osteoporosis or low-trauma fracture in the young, and type 2 diabetes mellitus [13]. Along with checking gonadal hormones for screening purposes in patients with HIV-associated weight loss, it is also important to have baseline levels before the initiation of MA in any patient in order to monitor testosterone levels after initiation.

Our patient is a young man with HIV-associated weight loss who was receiving MA. He presented with classic symptoms of androgen deficiency and prompt tapering of MA resolved his symptoms and normalized hormone levels, thus proving MA as the culprit for his hypogonadism rather than HIV itself. However, not all cases with hypogonadism will be symptomatic as is also the case with AI. Silent hypogonadism may be unnoticed until long-term consequences manifest as osteoporotic fractures or sarcopenia. Silent AI may also be a disaster waiting to occur in the form of overt AI if left undetected. Therefore, monitoring both the HPA and gonadal axis should be strongly considered in patients receiving MA.

## 4. Conclusion

MA is a medication often prescribed for anorexia and cachexia, but many prescribers may not recognize its adverse effects including AI and hypogonadism. Thus, clinicians must be aware of the potential HPA/HPG axes suppression induced by MA and take necessary measures to detect the problem and avoid detrimental consequences.

## References

[1] Ruiz Garcia V., López-Briz E., Carbonell Sanchis R., Gonzalvez Perales J. L., Bort-Marti S. (2013). Megestrol acetate for treatment of anorexia-cachexia syndrome. *Cochrane Database of Systematic Reviews*.

[2] Mann M., Koller E., Murgo A., Malozowski S., Bacsanyi J., Leinung M. (1997). Glucocorticoidlike activity of megestrol: a summary of food and drug administration experience and a review of the literature. *JAMA Internal Medicine*.

[3] Dev R., Del Fabbro E., Bruera E. (2007). Association between megestrol acetate treatment and symptomatic adrenal insufficiency with hypogonadism in male patients with cancer. *Cancer*.

[4] Delitala A. P., Fanciulli G., Maioli M., Piga G., Delitala G. (2013). Primary symptomatic adrenal insufficiency induced by megestrol acetate. *The Netherlands Journal of Medicine*.

[5] Kontula K., Paavonen T., Luukkainen T., Andersson L. C. (1983). Binding of progestins to the glucocorticoid receptor. *Biochemical Pharmacology*.

[6] Leinung M. C., Liporace R., Miller C. H. (1995). Induction of adrenal suppression by megestrol acetate in patients with AIDS. *Annals of Internal Medicine*.

[7] Nanjappa S., Thai C., Shah S., Snyder M. (2016). Megestrol acetate-Induced adrenal insufficiency. *Cancer Control*.

[8] McKone E. F., Tonelli M. R., Aitken M. L. (2002). Adrenal insufficiency and testicular failure secondary to megestrol acetate therapy in a patient with cystic fibrosis. *Pediatric Pulmonology*.

[9] Rochira V., Guaraldi G. (2014). Hypogonadism in the HIV-Infected Man. *Endocrinology and Metabolism Clinics of North America*.

[10] Bodenner D. L., Medhi M., Evans W. J., Sullivan D. H., Liu H., Lambert C. P. (2005). Effects of megestrol acetate on pituitary function and end-organ hormone secretion: A post hoc analysis of serum samples from a 12-week study in healthy older men. *American Journal of Geriatric Pharmacotherapy*.

[11] Geller J., Albert J., Geller S. (1982). Acute therapy with megestrol acetate decreases nuclear and cytosol androgen receptors in human BPH tissue. *The Prostate*.

[12] Lambert C. P., Sullivan D. H., Freeling S. A., Lindquist D. M., Evans W. J. (2002). Effects of Testosterone Replacement and/or Resistance Exercise on the Composition of Megestrol Acetate Stimulated Weight Gain in Elderly Men: A Randomized Controlled Trial. *The Journal of Clinical Endocrinology & Metabolism*.

[13] Bhasin S., Cunningham G. R., Hayes F. J. (2010). Testosterone therapy in men with androgen deficiency syndromes: an endocrine society clinical practice guideline. *The Journal of Clinical Endocrinology & Metabolism*.

